# Diagnosis and activity prediction of SLE based on serum Raman spectroscopy combined with a two-branch Bayesian network

**DOI:** 10.3389/fimmu.2025.1467027

**Published:** 2025-03-10

**Authors:** Qianxi Xu, Xue Wu, Xinya Chen, Ziyang Zhang, Jinrun Wang, Zhengfang Li, Xiaomei Chen, Xin Lei, Zhuoyu Li, Mengsi Ma, Chen Chen, Lijun Wu

**Affiliations:** ^1^ Department of Rheumatology and Immunology, People’s Hospital of Xinjiang Uygur Autonomous Region, Urumqi, China; ^2^ Xinjiang Clinical Research Center for Rheumatoid Arthritis, Urumqi, China; ^3^ College of Medicine, Shihezi University, Shihezi, China; ^4^ Xinjiang Medical University, Urumqi, China; ^5^ School of Computer Science and Technology, Xinjiang University, Urumqi, China; ^6^ College of Software, Xinjiang University, Urumqi, China; ^7^ College of Life Science and Technology, Xinjiang University, Urumqi, China

**Keywords:** systemic lupus erythematosus (SLE), Raman spectroscopy, diagnosis and prediction model, Bayesian net, autoimmune disease (AD)

## Abstract

**Objective:**

This study aims to examine the impact of systemic lupus erythematosus (SLE) on various organs and tissues throughout the body. SLE is a chronic autoimmune disease that, if left untreated, can lead to irreversible damage to these organs. In severe cases, it can even be life-threatening. It has been demonstrated that prompt diagnosis and treatment are crucial for improving patient outcomes. However, applying spectral data in the classification and activity assessment of SLE reveals a high degree of spectral overlap and significant challenges in feature extraction. Consequently, this paper presents a rapid and accurate method for disease diagnosis and activity assessment, which has significant clinical implications for achieving early diagnosis of the disease and improving patient prognosis.

**Methods:**

In this study, a two-branch Bayesian network (DBayesNet) based on Raman spectroscopy was developed for the rapid identification of SLE. Serum Raman spectra samples were collected from 80 patients with SLE and 81 controls, including those with dry syndrome, undifferentiated connective tissue disease, aortitis, and healthy individuals. Following the pre-processing of the raw spectra, the serum Raman spectral data of SLE were classified using the deep learning model DBayes. DBayesNet is primarily composed of a two-branch structure, with features at different levels extracted by the Bayesian Convolution (BayConv) module, Attention module, and finally, feature fusion performed by Concate, which is performed by the Bayesian Linear Layer (BayLinear) output to obtain the result of the classification prediction.

**Results:**

The two sets of Raman spectral data were measured in the spectral wave number interval from 500 to 2000 cm-1. The characteristic peaks of serum Raman spectra were observed to be primarily located at 1653 cm^-1^ (amide I), 1432 cm^-1^ (lipid), 1320 cm^-1^ (protein), 1246 cm^-1^ (amide III, proline), and 1048 cm^-1^ (glycogen). The following peaks were identified: 1653 cm^-1^ (amide), 1432 cm^-1^ (lipid), 1320 cm^-1^ (protein), 1246 cm^-1^ (amide III, proline), and 1048 cm^-1^ (glycogen). A comparison was made between the proposed DBayesNet classification model and traditional machine and deep learning algorithms, including KNN, SVM, RF, LDA, ANN, AlexNet, ResNet, LSTM, and ResNet. The results demonstrated that the DBayesNet model achieved an accuracy of 85.9%. The diagnostic performance of the model was evaluated using three metrics: precision (82.3%), sensitivity (91.6%), and specificity (80.0%). These values demonstrate the model’s ability to accurately diagnose SLE patients. Additionally, the model’s efficacy in classifying SLE disease activity was assessed.

**Conclusion:**

This study demonstrates the feasibility of Raman spectroscopy combined with deep learning algorithms to differentiate between SLE and non-SLE. The model’s potential for clinical applications and research value in early diagnosis and activity assessment of SLE is significant.

## 1 Introduction

Systemic lupus erythematosus (SLE) is a chronic autoimmune disease that can affect a wide range of tissues and organs, exhibiting significant clinical heterogeneity (1). While the long-term prognosis of patients with SLE has improved markedly in recent years, irreversible chronic organ and tissue damage caused by the disease remains a significant challenge (2). A sustained state of disease activity is an important predictor of organ damage and mortality (3). It is therefore crucial to facilitate an early and accurate diagnosis of SLE, coupled with regular monitoring of disease activity. This enables the development and adjustment of individualized treatment plans, as well as the control of disease activity, with the ultimate goal of improving patient prognosis. At present, the diagnosis of SLE is primarily based on the classification criteria set forth by the European League Against Rheumatism (EULAR) in 2019 (4), while the assessment of disease activity is predominantly conducted using instruments such as the SLICC/ACR Damage Index (SDI), the SLE Disease Activity Index 2000 (SLEDAI-2K) (5), and the Systemic Lupus Erythematosus Disease Activity Score (SLE-DAS) (6). These assessment tools integrate a range of data points, including clinical symptoms, signs, laboratory markers, organ involvement, and the physician’s subjective assessment. However, the complexity and heterogeneity of the clinical manifestations of SLE present significant challenges to the diagnosis and assessment of disease activity. Firstly, the selection of assessment tools is somewhat variable, and the results are susceptible to subjective factors and a lack of clinical experience among physicians, which may result in early misdiagnosis or underdiagnosis. Furthermore, although anti-dsDNA and anti-SM antibodies demonstrate high specificity in the diagnosis of SLE, the sensitivity of anti-SM antibodies is relatively low (approximately 30% are positive), and these antibodies may also be positive in other connective tissue diseases (7). Furthermore, traditional clinical indicators are unable to reflect the immunopathological status or chronic damage of tissues and organs in real-time, resulting in an inaccurate assessment of disease activity and, consequently, delays in treatment (8). It is therefore imperative to develop a non-invasive, efficient, and accurate method for the early diagnosis and activity assessment of SLE.

Raman spectroscopy is a molecular vibration-based scattering spectroscopy technique that enables the non-destructive detection of biomolecules, including proteins, enzymes, and nucleic acids. The vibrational frequency of the sample reflects its phenotypic ‘fingerprint’ and physiological and biochemical status (9). Owing to its non-destructive and rapid nature, Raman spectroscopy in conjunction with artificial intelligence (AI) technology has been employed with increasing frequency in the field of medical diagnosis. Prior research has demonstrated the efficacy of machine learning models in the classification and diagnosis of autoimmune diseases. Xiaomei Chen et al. employed a combination of Raman spectroscopy and a PSO-SVM algorithm to facilitate the classification and diagnosis of patients with primary desiccation syndrome, achieving an accuracy rate of 94.44% (10). Xue Wu et al. successfully implemented a rapid diagnosis of patients with pSS-ILD through the utilization of serum Raman spectroscopy and machine learning algorithms (11). Chen Chen et al. proposed a hybrid sampling technique, R-GDORUS, to address the issue of data imbalance in medical Raman spectroscopy studies (12). However, traditional machine learning models typically necessitate time-consuming data preprocessing, which may result in the loss of band-related information and an increased risk of overfitting (13). Deep learning, an important branch of machine learning, is adept at extracting salient features from complex data, particularly in the domains of image and signal analysis (14).

In recent years, Raman spectroscopy in conjunction with deep learning has been extensively employed in the investigation of tumors, infectious diseases, and other related fields (15). Wei Shua and colleagues have demonstrated the potential of Fourier Transform Infrared Spectroscopy (FTIR) and a deep learning model for the early diagnosis of rheumatoid arthritis and ankylosing spondylitis (16). Cheng Ningtao and colleagues developed a classification model through spectroscopy-based deep learning and proposed a nano-plasma biosensor chip (NBC) platform for rapid screening of liver cancer and other types of cancers (17). Vijayakumar Selvarani et al. employed a combination of Raman spectroscopy and a deep learning model to facilitate the diagnosis and staging of breast cancer (18). Lianyu Li et al. utilized a deep neural network architecture to develop four distinct multi-task network models for oral cancer, to achieve intelligent diagnosis through the simultaneous processing of multiple classification tasks, including tumor staging, lymph node staging, and histological grading. The accuracy of these models reached 83.0% (19). It has been demonstrated that the combination of Raman spectroscopy with deep learning has the potential to facilitate the early diagnosis and prediction of the activity of chronic diseases. However, in classification studies of SLE and other connective tissue diseases, the models are susceptible to overfitting due to the overlap of different disease features and the overlap of spectral vibrational peaks. Furthermore, the high degree of consistency in the pathological features of SLE presents a significant challenge in feature extraction for activity assessment, which in turn limits the effectiveness of classification and diagnosis.

In order to address the aforementioned issues, this study proposes a novel model, the dual-branch Bayesian network (DBayesNet), which combines the dual-branch structure with Bayesian ideas. The dual-branch structure enables the handling of disparate tasks through the utilization of two independent branches, facilitating the extraction of features from varying perspectives. This enhances the model’s capacity for representation and renders it well-suited for the extraction and classification of intricate disease characteristics. Xiaopu He et al. put forth a novel two-branch lesion-aware neural network to categorize intestinal lesions, delving into the intrinsic relationship between diseases to enhance the efficacy of colon disease classification (20). Xinya Chen et al. advanced an enhanced two-branch attention network to expeditiously identify diabetic nephropathies, integrating the fusion of shallow and deep features, local and global features, and improving the model’s classification accuracy (21). It is therefore essential to construct an excellent classification model in order to facilitate disease recognition. In this study, the Bayesian approach is further introduced to enhance the robustness and generalization ability of the model, combining the advantages of feature extraction from the two-branch structure. The Bayesian approach effectively addresses the issue of insufficient sample size by quantifying parameter uncertainty and integrating prior knowledge with sample data, thereby enhancing the accuracy of prediction and classification and providing more rapid and reliable support for disease diagnosis.

In conclusion, this paper proposes a model called the dual-branch Bayesian network (DBayesNet), which combines the uncertainty quantification ability of Bayesian ideas with the feature extraction advantage of the dual-branch structure. The incorporation of the Bayesian idea enhances the interpretability of the model results, while the dual-branching structure improves the model’s representational ability. This addresses the issue of poor classification due to the high similarity of disease features in the complexity of connective tissue disorders and the assessment of SLE activity. The DBayesNet model is applied to classify and diagnose SLE and non-SLE, and the experimental results demonstrate that its accuracy is superior to that of the other eight traditional classification models. Additionally, the DBayesNet model demonstrated considerable success in the assessment of SLE patient activity, substantiating the viability of serum Raman spectroscopy in conjunction with deep learning algorithms for the diagnosis and prediction of SLE activity. This study presents an efficient and accurate assessment and prediction strategy for the early diagnosis and screening of SLE.

## 2 Materials and methods

### 2.1 Study population and sample preparation

A total of 161 subjects, including 80 patients with systemic lupus erythematosus (SLE), were enrolled in the study at the Department of Rheumatology and Immunology of Xinjiang People’s Hospital between 2022 and 2023. The participants were classified as having mild, moderate, or severe disease activity according to the SLE Disease Activity Index 2000 (SLEDAI-2K) activity level. The onset of SLE is characterized by a higher prevalence in women of childbearing age; therefore, to avoid any potential gender imbalance in the control group, a total of 81 age- and gender-matched serum samples from the same period of time were included, comprising patients with dry syndrome, undifferentiated connective tissue disease, and aortitis, as well as healthy individuals. All blood samples were obtained from peripheral veins without the use of anticoagulant agents. The samples were subjected to centrifugation at 4°C and 4000 rpm at high speed. Following a 10-minute centrifugation period, a top clarification was obtained and stored in a refrigerator at -80°C. Following the thawing of the serum, Raman spectroscopic signals were collected.

1. Inclusion criteria: Patients with SLE who have been definitively diagnosed in accordance with the criteria set forth by the European League Against Rheumatism (EULAR) in 2019 (4); patients who have been diagnosed with dry syndrome in accordance with the classification criteria established by the American College of Rheumatology/European League Against Rheumatism (ACR/EULAR) in 2016 (22). A diagnosis of undifferentiated connective tissue disease in accordance with the classification criteria for undifferentiated connective tissue disease proposed in 1998 (23); and a diagnosis of aortic inflammation in accordance with the 2022 classification criteria developed jointly by the ACR and the European League Against Rheumatism (EULAR) joint classification criteria for the diagnosis of aortitis (24). The SLEDAI-2K (Systemic Lupus Erythematosus Disease Activity Index-2000) (25) scale allows for the categorization of disease activity as follows: low activity is indicated by a SLEDAI score of ≤ 6, moderate activity by a score of ≥ 7 and ≤ 12, and severe activity by a score of > 12.
2. Exclusion criteria: patients with malignant neoplasms, diabetes mellitus, other rheumatic and immune diseases, and other systemic disorders.

### 2.2 Data collection and spectral analysis

Use a pipette to aspirate 15 μL of the sample and drop it on aluminum foil. After drying at room temperature, the Raman signal was measured directly. A high-resolution confocal Raman spectrometer (LabRAM HR Evolution, gora Raman Spectroscopy, ideal optics, China) was used with a YAG laser excitation wavelength of 785 nm, a 10× objective lens, an integration time of 15 s, a laser power of 160 mW, and a continuous acquisition mode. Raman spectra of serum samples were measured in the range of 500-2000 cm, and three spectral signals were recorded from different positions of each sample.

### 2.3 Data preprocessing

As the serum Raman spectra collected by the spectrometer are interfered with by factors such as measurement conditions, detection environment, and hardware facilities, the spectral data are too complicated, which will affect the analysis effect to a large extent. Therefore, it is necessary to perform pre-processing operations on the acquired Raman spectral data. For the raw spectra, we perform band selection, smoothing, baseline correction, and then outlier removal and normalization preprocessing. Firstly, the 500cm^-1^ to 2000cm^-1^ band of the Raman spectrum was selected, which belongs to the fingerprint region of the Raman spectrum, so this band range was used for analysis. The collected serum spectral data were smoothed and filtered using the Savitzky-Golay (S-G) algorithm, and the final selection of the moving window size was 9 and the polynomial order was 2. The baseline correction used the adaptive iterative reweighted penalized least squares (airPLS) algorithm to fit the fluorescence background signals of the Raman spectra, which can effectively remove the background noise in the Raman spectra and improve the quality of the spectral data, thus improving the quality of the spectral data for the subsequent analysis. Data quality, thus providing more accurate information for subsequent analyses. Finally, we normalize the integrated area under the preprocessed Raman spectral curve and scale the eigenvalues to reduce the data complexity and improve the model convergence speed.

### 2.4 DBayesNet model

**Figure 1:**
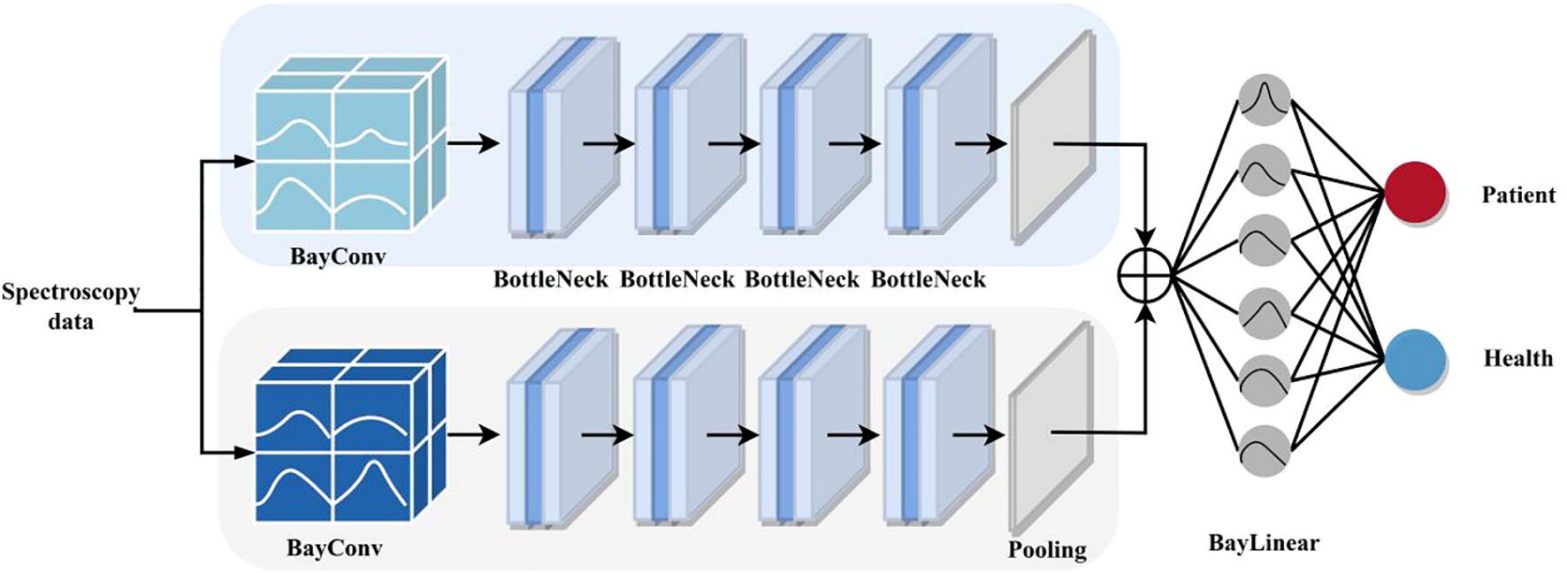
DBayesNet network diagram.

As shown in **Figure 1**, DBayesNet is mainly composed of a two-branch structure, each branch consists of a Bayesian Convolution (BayConv) module, BottleNeck (BottleNeck) module, and AdaptiveAvgPool, of which the BottleNeck module consists of a total of four BottleNeck layers, where the parameters of two branches are set differently to extract different levels of features, and then the features of the upper and lower branches are fused by Concate, through the Bayesian Linear layer (BayLinear). The parameters of the two branches are set differently to extract features at different levels, and then the features of the upper and lower branches are fused by Concrete and output through the Bayesian Linear layer (BayLinear) to get the classification prediction results.

In DBayesNet, the innovation of BayConv and BayLinear is the introduction of the ability to estimate uncertainty. While traditional convolutional and linear layers learn only deterministic values of weights, Bayesian neural networks are able to learn the uncertainty of the weights so that instead of a single deterministic value, the weights obey some kind of probability distribution. This ability allows BayConv and BayLinear to provide probabilistic information for prediction rather than just a single classification or regression output. In addition, BayConv and BayLinear are able to output uncertainty estimates at the time of prediction, which helps the model to better deal with situations with high uncertainty and improves the reliability and applicability of the model. Especially on small sample datasets, BayConv and BayLinear are able to use uncertainty information to reduce the risk of overfitting and improve the generalization ability of the model. In summary, the introduction of BayConv and BayLinear enables DBayesNet to handle uncertainty and small-sample data more flexibly, providing more comprehensive and reliable probabilistic information for model prediction, thus improving the performance and application value of the model. The parameter settings of BayConv and BayLinear are as follows **Table 1** shows.

**Table 1:** Parameter settings for BayConv and BayLinear.

Assemblies	Prior distribution Initial mean	Prior distribution Standard deviation	Posterior distribution Initial mean	Posterior distribution Logarithmic variance
BayConv	0	0.1	(0, 0.1)	(3, 0.1)
BayLinear	0	0.1	(0, 0.1)	(3, 0.1)

### 2.5 Feature extraction

In DBayesNet, BayConv can provide uncertainty estimates for each feature extraction process. This means that at a shallow level, the model learns not only to extract features but also the uncertainty of those features. This uncertainty-guided feature extraction helps the model to better understand the noise and variations in the data and thus make more reliable predictions. We can then extract features at different scales by using different configurations of BayConv in both branches, capturing multi-level information in the data. At a deeper level, features from both branches are fused and classified by BayLinear, which takes into account not only the values of the fused features but also their uncertainty. This probabilistic feature fusion helps the model to take into account the reliability of the features when making decisions, thus improving the accuracy and robustness of the predictions. With the two-branch architecture, uncertainty can be passed from shallow to deep levels and integrated into the final decision. This helps the model to make more reasonable predictions in the face of high uncertainty.

In the DBayesNet model, the two branches employ convolutional kernels of different sizes or different pooling strategies to extract features at different scales. This multi-scale feature extraction helps the model to capture different levels of information in the data, and fusion at a deeper level can achieve complementary features, make more reasonable predictions in the face of higher uncertainty, and enable the model to capture more complex data patterns. Improve the generalization ability of the model. Overall, by combining BayConv and BayLinear, the two-branch structure is not only able to extract features of different scales and perspectives, but also able to fuse the probabilistic information of these features at a deep level, thus improving the prediction accuracy and robustness of the model.

### 2.6 Modeling indicators

The performance of the classification model in this study was assessed by sensitivity, precision, specificity, and accuracy as in Equations 1-4), the confusion matrix is shown in **Table 2** shown.

**Table 2:** Confusion matrix.

Actual		
Predicted	Positive	Negative
Positive Negative	TP FN	FP TN


(1) $$Sensitivity=\frac{TP}{TP+FN}$$

(2) $$Precision=\frac{TP}{TP+FP}$$

(3) $$Specificity=\frac{TN}{TN+FP}$$

(4) $$Accuracy=\frac{TP+TN}{TP+TN+FP+FN}$$

## 3 Results and discussion

### 3.1 Population and clinical characteristics

**Table 3** presents the data regarding the age and gender of the SLE patients and controls. The 80 patients with systemic lupus erythematosus (SLE) were categorized into three activity classes according to the SLEDAI-2K criteria based on their symptoms and clinical indicators. The number of cases exhibiting mild, moderate, and severe activity levels were 44, 30, and 6, respectively.

**Table 3:** Summary information on age and sex of patients and healthy controls.

	SLE (N=80)	Non-SLE (N=81)	significant P-value
Age			
	43.6 ± 13.9	46.9 ± 11.4	P<0.05
Gender			
Male	11	9	P>0.05
Female	69	71	

### 3.2 Spectral analysis

**Figure 2:**
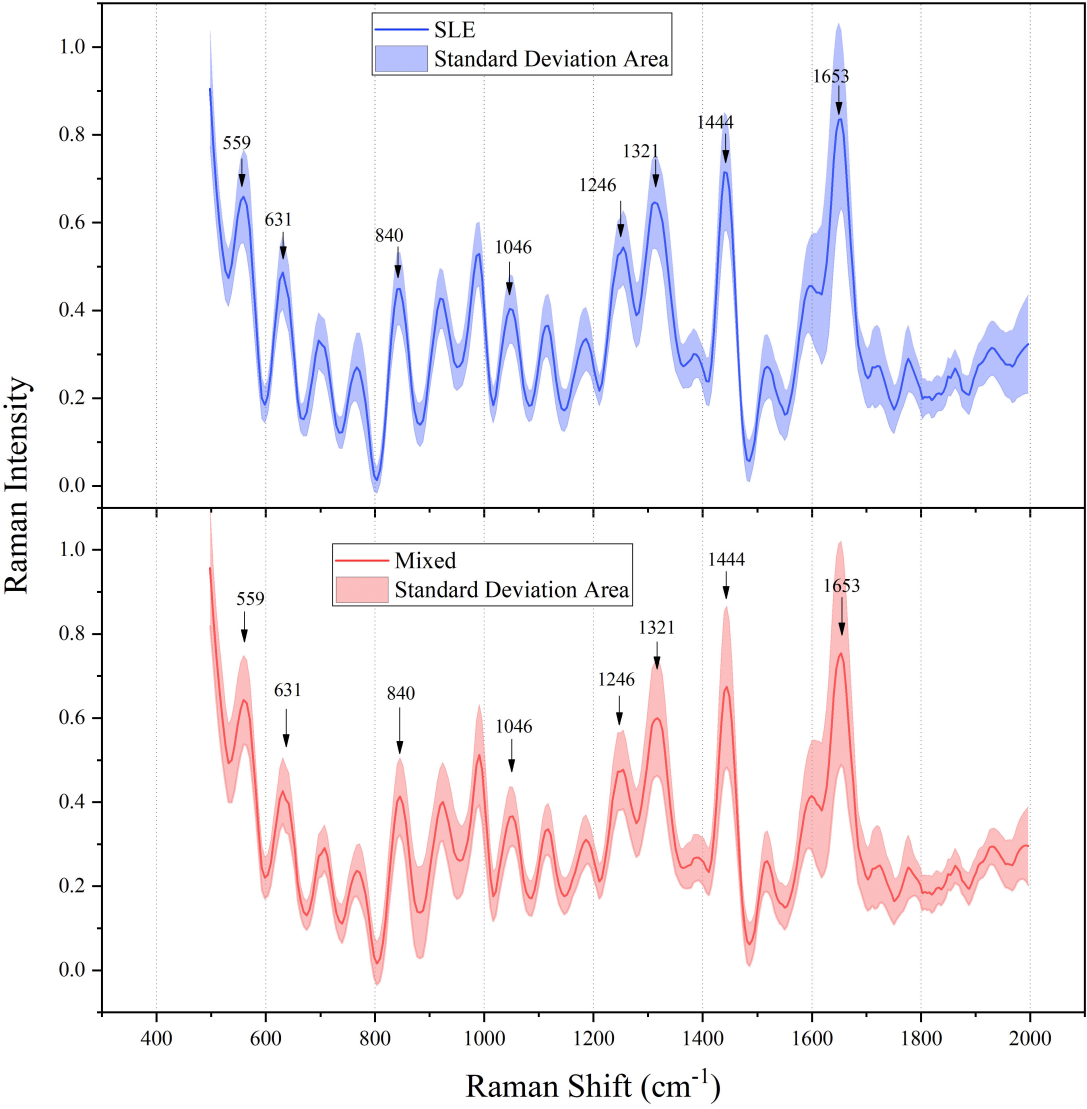
Normalized mean Raman spectra of serum samples from SLE patients and controls.

**Figure 2** shows the average preprocessed Raman spectra of serum samples from SLE patients and controls in the range of wave numbers 500 to 2000 cm^-1^. As shown, the spectral peaks of the spectra of SLE and the control group containing other connective tissue diseases and healthy people were similar, and the difference between the two was the magnitude of the peaks of the curve fluctuations. The characteristic peaks of the serum Raman spectra were mainly at 559 cm^-1^, 631 cm^-1^, 700 cm^-1^, 840 cm^-1^, 1046 cm^-1^, 1246 cm^-1^, 1320 cm^-1^ and 1653 cm^-1^. The corresponding molecular information of the Raman peaks is listed in **Table 4**. The strongest peak is at 1653 cm^-1^ (Amide I), which is mainly caused by the carbon-oxygen (C=O) stretching vibration of the peptide bond, and the position and shape of the Amide I band in the β-folded structure can provide important information about the protein structure (26). The characteristic peak at 1432 cm^-1^ (CH2 scissoring vibration) is usually associated with the bending vibration of the methyl group (-CH2-) in lipid molecules, and this vibration mode can provide important information about the internal structure of lipid molecules and intermolecular interactions (27); 1320 cm^-1^ is the region of the protein corresponding to the guanine base, which involves the bending or deformation of the carbon-hydrogen bond (CH), and this change is reflected in the infrared (IR) and Raman spectra and can provide important information about the structure and dynamics of proteins (28, 29);1246 cm^-1^ represents the amide III and CH2 wobble vibrations, which in vibrational spectroscopic studies of proteins are involved in the CH2 groups of the Glycine backbone and Proline side chain of proteins (30). 1046 cm^-1^ corresponds to a higher amount of glycogen than the non-SLE population. Glycogen not only serves as a glucose reservoir but also provides antioxidant defenses through the production of NADPH and subsequently reduced glutathione, implying that glycogen exerts important biological effects through metabolic pathways that are of value in inflammatory responses and metabolic regulation (31). 840 cm^-1^ contains the Tyrosine ring breathing vibration (TBR), a vibrational pattern whose changes can reflect direct and indirect interactions with the charge distribution of surrounding solvent molecules, indirectly detecting changes in the protein microenvironment (32). The C-C torsion vibration in Raman spectroscopy may be a useful probe for the study of aromatic compounds due to the activity of the C-C torsion vibration in Raman spectra (33).

**Table 4:** Peak positions and tentative assignments of the main spectral bands.

Wavenumber (cm)^-1^	Corresponding substance
1653	Amide I (β-sheet) Amide I band (protein band)
1432	CH_2_ scissoring vibration (lipid band)
1425	Deoxyribose (B, Z-marker)
1320	G (DNA/RNA); CH deformation (proteins)
1246	Amide III and CH_2_ wagging vibrations from glycine backbone and proline side chains
1260	Amide III (protein band); Protein band
1048	Glycogen
852	Tyrosine ring breathing; Glycogen
720	DNA
631	C-C twist aromatic ring
559	OH out-of-plane bending

**Figure 3:**
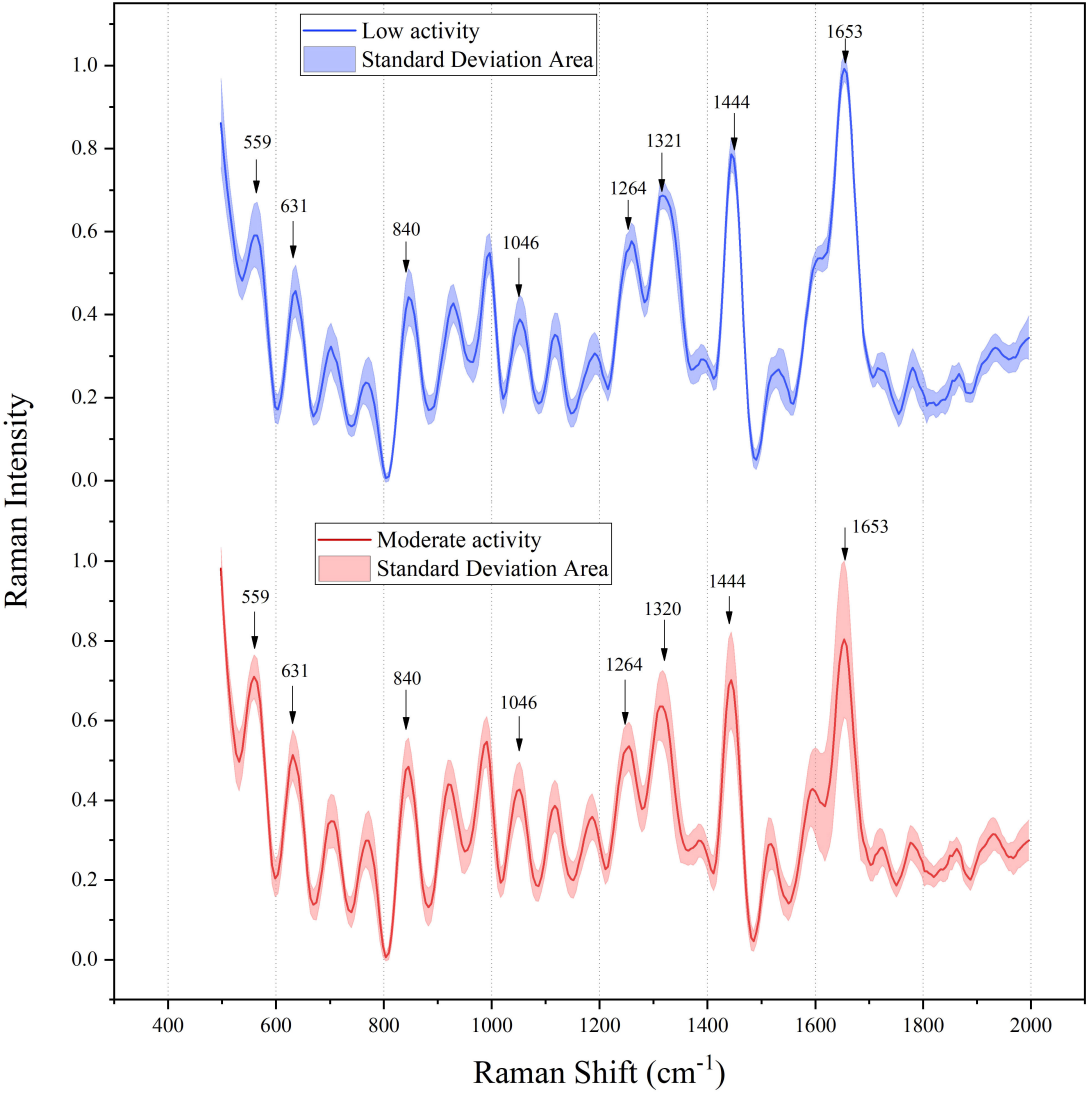
Mean preprocessed Raman spectra of SLE patients with different activity levels.

In addition, **Figure 3** shows the average preprocessed Raman spectra of SLE patients with different activity levels in the range of wave numbers from 500 to 2000 cm^-1^. The characteristic peaks of the serum spectra were mainly at 559 cm^-1^, 631 cm^-1^, 1260 cm^-1^, 1444 cm^-1^, and 1653 cm^-1^, and the magnitude of the peaks of curve fluctuations varied with different activities, as shown in **Table 4** corresponding to protein, deoxyribose, and amino acids, respectively. The above spectra showed the differences in chemical substances in the serum of different patients, which provided an important theoretical basis for the subsequent classification study.

### 3.3 Classification model results

#### 3.3.1 Disease diagnosis

**Figure 4:**
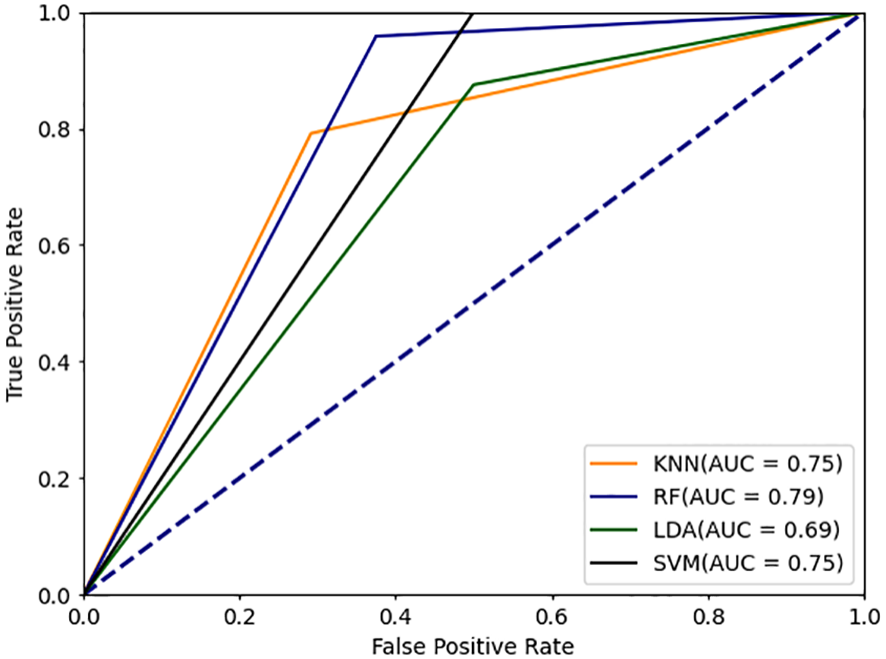
ROC curve for machine learning classification model.

**Figure 5:**
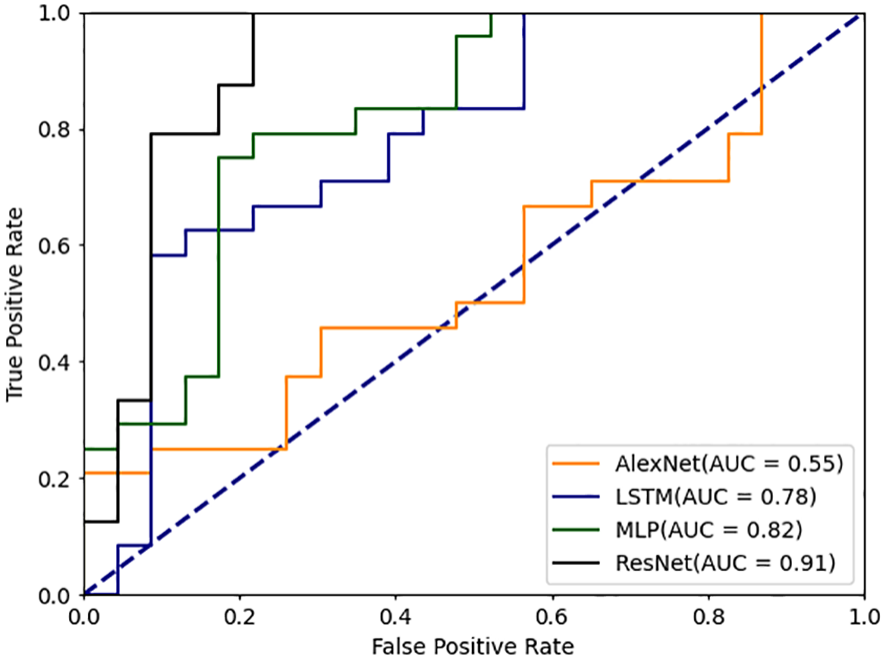
ROC curve for deep learning classification model.

In this study, we propose a novel deep learning model-DBayesNet, which achieves significant results in experiments to distinguish systemic lupus erythematosus (SLE) from dry syndrome (SS), thyroid disease (TA), undifferentiated connective tissue disease (UCTD), and healthy controls (HC). As **Table 5** Comparison of experimental results with traditional machine learning and deep learning models shown, DBayesNet achieves the best classification results in terms of sensitivity, specificity, precision, and accuracy, with values of 91.6%, 80.0%, 82.3%, and 85.9%, respectively. In addition, ROC curves of different models are shown (**Figures 4**, **5**), the area under the ROC curve represents the AUC value, which can be used to quantify the accuracy of the classifier; the larger the AUC value, the more reliable the model is. The results of the experimental study demonstrate that the DBayesNet model, developed in this study, is an optimal tool for classifying SLE versus non-SLE populations.

**Table 5:** Comparison of experimental results with traditional machine learning and deep learning models.

Model	Accuracy	Precision	Sensitivity	Specificity
KNN	0.8458	0.72	0.925	0.667
SVM	0.7416	0.74	0.82	0.59
RF	0.8333	0.82	0.72	0.48
LDA	0.75	0.75	0.69	0.42
ANN	0.7234	0.7234	1.00	0.43
AlexNet	0.53	0. 53	0.67	0.39
LSTM	0.7	0. 67	0.83	0.57
resnet	0.85	0.81	0.92	0.78
**DBayesNet**	**0.8593**	**0.8228**	**0.9164**	**0.7999**

Bold values indicate that DBayesNet achieves optimal classification in terms of sensitivity, specificity, precision, and accuracy.

#### 3.3.2 Assessment of disease activity

In this study, to further validate the performance of the model in classifying the activity level of SLE patients, a classification task was performed for 76 SLE patients with activity level, and the results are shown in **Table 6** shown, DBayesNet achieved the best results for its sensitivity, specificity, precision, and accuracy, especially the accuracy was as high as 93.3%, which was significantly better than the other models involved in the comparison, and secondly, DBayesNet’s sensitivity reached 97%, which demonstrated that DBayesNet was able to identify the true positive cases more accurately. Compared with traditional machine learning models such as KNN, SVM, RF, and LDA, DBayesNet improves the accuracy of activity classification by 2.03%, 19.42%, 6.37%, and 11.59%, respectively, and this result fully demonstrates the powerful ability of the DBayesNet model in the task of classifying disease activity. Meanwhile, DBayesNet also improves the accuracy of activity classification by 2.39%, 6.94%, 2.42%, and 2.42%, respectively, compared with deep learning models such as ANN, AlexNet, LSTM, and MLP, which once again proves the superiority of DBayesNet in the field of deep learning.

**Table 6:** Comparison of model effectiveness in classifying disease activity.

Model	Accuracy	Precision	Sensitivity	Specificity
KNN	0.8178	0.84	0.84	0.8
SVM	0.7416	0.42	0.42	0.94
RF	0.87	0.75	1.00	0.79
LDA	0.75	0.95	0.95	0.73
ANN	0.91	0.89	0.89	0.92
AlexNet	0.82	0. 86	0.67	0.92
LSTM	0.91	0. 89	0.89	0.92
resnet	0.91	0.82	1.00	0.85
**DBayesNet**	**0.933**	**0.85**	**0.97**	**0.92**

Bold values indicate that DBayesNet achieves optimal classification in terms of sensitivity, specificity, precision, and accuracy.

**Figure 6:**
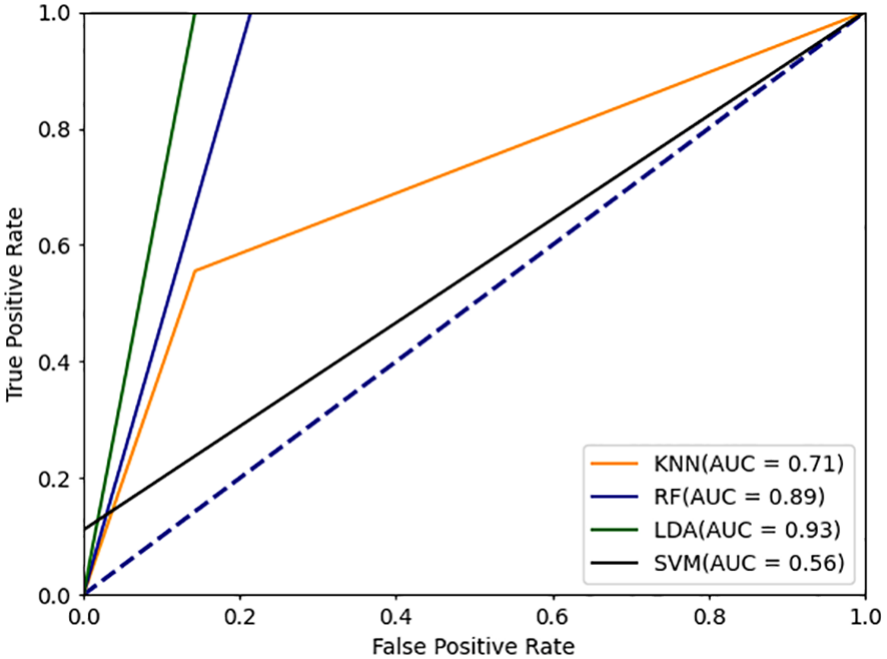
ROC curve for machine learning classification model.

**Figure 7:**
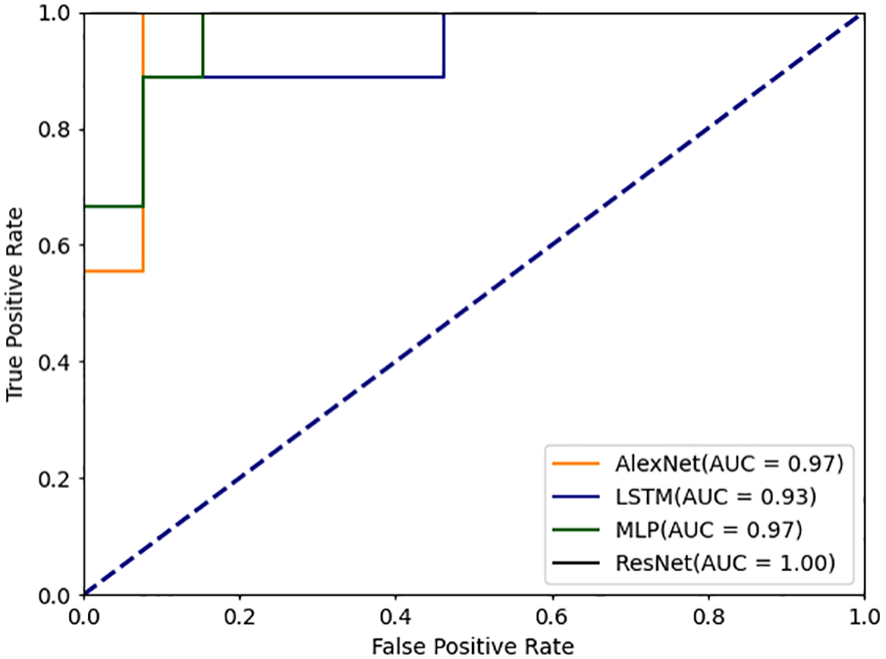
ROC curve for deep learning classification model.

In addition, by comparing the ROC curves of different models (**Figures 6**, **7**), the advantage of the DBayesNet model in classification performance can be visualized. The ROC curves show that the DBayesNet model has high sensitivity while maintaining high specificity, which makes it more accurate in determining the disease activity of SLE patients in clinical applications and provides a more reliable diagnosis basis for doctors.

## 4 Discussion

SLE is a chronic, multi-system autoimmune disease characterized by widespread inflammation and tissue damage, with a complex and varied clinical picture, a prolonged and recurrent course, and an increased risk of organ damage and death as disease activity increases. Early and accurate diagnosis and reduction of disease severity and inflammatory response, as well as minimizing the use of hormones and immunosuppressive drugs, can improve the long-term prognosis of patients. Currently, the diagnostic and activity scoring criteria widely used in clinical practice are influenced by many external factors. Therefore, there is an urgent need for an objective and accurate test for rapid diagnosis and activity assessment, especially in the early stages of the disease. Vibrational spectroscopy, as a non-invasive assay, closely correlates changes in spectral peaks with disease-specific biochemical changes, which makes spectroscopy a powerful tool for early diagnosis and classification of diseases at the molecular level. In this study, we propose a two-branch Bayesian network structure that compensates for the high degree of overlap of spectroscopy in connective tissue diseases for rapid and accurate serological differentiation of SLE from other non-SLE populations, as well as for activity assessment of SLE patients. By comparing with traditional machine learning and deep models (KNN, SVM, RF, LDA, ANN, AlexNet, LSTM, MLP), the two-branch Bayesian model proposed in this study shows stronger classification ability in disease diagnosis and activity assessment. Specifically, the classification accuracy of DBayesNet was improved by 1.43%, 11.76%, 2.6%, and 10.96%, respectively, compared to the traditional model, and this result fully demonstrates the powerful ability of the DBayesNet model in disease diagnosis tasks, and the classification accuracy of the DBayesNet model was improved by 13.59%, respectively, compared to other deep learning models, 7.21%, 5.08% and 9.33%, further proving DBayesNet’s advancement in the field of deep learning. DBayesNe’s high accuracy of 85.9% indicates that the model possesses excellent prediction performance on the overall samples; secondly, the accuracy of 82.3% implies that the instances predicted to be positive samples by DBayesNet are truly positive samples in a proportion is high, which significantly reduces the misdiagnosis rate; the sensitivity of 91.6% reflects the high reliability of the model in identifying SLE patients; and finally, the specificity of 80.0% indicates that DBayesNet also exhibits good accuracy in identifying non-SLE patients. Furthermore, the DBayesNet model demonstrated the capacity to accurately differentiate between the various activity levels of SLE. Its results were found to be consistent with the clinical assessment of disease activity in 76 SLE patients, thereby confirming the effectiveness of the model in classifying SLE activity.

Based on the spectrograms, it can be seen that some of the peaks of the SLE and non-SLE populations are close to each other, indicating that the two sera have similar biomolecules, and the differences are reflected in the size of the characteristic peaks of the two groups, e.g., 1653 cm^-1^ (Amide I), 1432 cm^-1^ (Lipids), 1320 cm^-1^ (Proteins with guanine bases), 1246 cm^-1^ (Amide III, Proline), 1048 cm^-1^ (Glycogen), 852 cm^-1^ (Tyrosine), 720 cm^-1^ (DNA), 631 cm^-1^ (Aromatic compounds), and others. In previous studies, it has been found that when immune cells are exposed to external stimuli, they promote the conversion of mechanical signals into responses within the immune cells, and this regulatory mechanism of the immune system may include the role of amide compounds in immune signaling (34). In SLE patients, the characteristic peak of activity at 1260 cm (Amide III) also includes amides, implying that monitoring amide levels in the serum of SLE patients could be useful in identifying the disease and predicting activity. From the spectral peak at 1432cm^-1^ Regarding the differences in lipid content, systemic lupus erythematosus (SLE) is associated with an increased risk of cardiovascular disease because in normal subjects high-density lipoproteins (HDL) can exert vasoprotective activity by promoting the activation of transcription factor 3 (ATF3), which results in the down-regulation of the inflammatory response induced by the Toll-like receptor (TLR), whereas in SLE patients HDL HDL in SLE patients exhibits significant ATF3-inducing and pro-inflammatory cytokines, and the loss of its anti-inflammatory effect ultimately leads to immune dysregulation (35). Croca et al. (2003) demonstrated that serum lipid profiles and abnormalities in autoantibody and T-lymphocyte responses to lipids may contribute to the development of atherosclerosis (36). Furthermore, they established a positive correlation between activity index (SLEDAI) scores in patients with SLE and an increased risk of cardiac damage. Consequently, the utilization of rapid and straightforward methods to monitor and regulate serum lipid levels in early life has significant implications for reducing the risk of atherosclerosis.

In this study, we also found that the levels of 1246 cm^-1^ (proline) and 852 cm^-1^ (lysine) in SLE patients were significantly different from those in the control group. YangJie et al. found that proline-rich tyrosine kinase 2 (Pyk2) may be positively correlated with the pathogenesis and disease activity of SLE through its involvement in the aberrant activation of lymphocytes and that patients with combined renal involvement had significantly higher levels of Pyk2 than those with other organ involvement. Were significantly higher in patients with combined renal involvement than in those with other organ involvement. In addition, lysine is involved in neurotransmission, hormone synthesis, and antioxidant defense, and has become a therapeutic target for various diseases. Differences in waveforms 720cm^-1^ and 1425cm^-1^ deoxyribose were found between the two groups in diagnostic classification and activity classification, which is similar to previous studies.DNA and its associated antibodies in serum play an important role in the diagnosis and treatment of SLE, and anti-DNA antibody is a marker of classification and disease activity, contributing to pathogenesis through the formation of immune complexes deposited in tissues or stimulating the production of cytokines (37), which means that the monitoring of antibody titers at the spectral level may help to precede preclinical diagnosis of the disease and inform the assessment of its activity and efficacy. Finally, the present study found differences in wave peaks at 1048 cm^-1^ (glycogen) between the two groups. Previous studies have shown that activated lymphocyte-derived DNA (ALD-DNA) drives macrophage polarization towards M2b, produces inflammatory cytokines and induces inflammation, and Zhao, Hanqing et al. performed glucose metabolomics analysis on ALD-DNA-stimulated macrophages and found that Enhanced gluconeogenesis (38), which is similar to the present spectral peak corresponding to more glycogen in SLE patients than controls, which further deepens our understanding of disease pathogenesis and provides clues for interventional exploration.

In this study, based on the differences in the characteristic peaks of serum Raman spectra between SLE and controls, which correspond to the differences in substances such as proteins, nucleic acids, glycogen, and lipids, we offer the possibility of using Raman spectra in combination with a deep learning classification model to differentiate SLE. The accuracy of DBayesNet proposed in this study for disease classification and diagnosis is as high as 85.93%, which is significantly higher than that of traditional machine learning and deep learning models, and we also applied the model to the activity level classification data of SLE patients, with an accuracy as high as 93.33%, which demonstrates a strong classification efficacy. In addition, we also analyzed the relevant substances corresponding to the characteristic peaks of the serum Raman spectra in the spectral data of SLE patients and controls, as well as the mild and moderate activity levels of SLE patients, which fully proved the possibility of the two-branch Bayesian model in the clinical practice, which can not only be used for detecting changes in serum proteins, nucleic acids, and lipids, but also be used for early diagnosis of the disease and assessment of the activity level, to achieve better diagnosis and treatment, and to achieve a more personalized diagnosis and treatment. It can also be used for early diagnosis and assessment of disease activity to provide personalized diagnosis and treatment and achieve a better long-term prognosis.

In addition to the application of Raman spectroscopy to immunological diseases explored in this study, techniques such as mass spectrometry and spectroscopy in assisted diagnosis have become a significant component of clinical medicine. Chen et al. explored the application of mass spectrometry and spectroscopy combined with machine learning (ML) in *in-vitro* diagnostics (IVD), which provides a new perspective in dealing with complex datasets and highlights the importance of multimodal analysis in comprehensively analyzing biological samples (39). Another study revealed the unique metabolic pattern of SLE patients by constructing a large-scale cohort using nano-assisted laser desorption/ionization mass spectrometry (LDI MS) technology and screening four potential metabolic biomarkers, which provided new insights into the pathological mechanisms and clinical diagnosis of SLE (40). In contrast, Wang et al. utilized Co3O4/C composites and LDI MS technology to expedite and cost-effective acquisition of metabolic fingerprints SMFs from pregnant women’s serum for the purpose of evaluating SLE activity. They further developed a diagnostic method employing machine learning to differentiate between patients with active SLE, thereby providing a novel instrument for precision medicine in SLE (41). The significance of metabolic fingerprinting technology and machine learning in the diagnosis of Systemic Lupus Erythematosus (SLE) and the discovery of biomarkers is demonstrated by these studies. These techniques can reveal metabolic differences between SLE patients and healthy individuals, which is critical for early diagnosis and management of the disease. The incorporation of technological advancements has been demonstrated to enhance diagnostic precision, whilst concomitantly reducing financial expenditure and alleviating patient burden.

Nevertheless, owing to the restricted sample size of the present study, its findings are subject to certain limitations. Furthermore, in the context of the study’s objective to examine the classification of patient activity, it is noteworthy that only six cases of severe activity were identified. This limitation arises from the consideration that the sample size was inadequate for the classification to be rendered comparable. Consequently, the decision was taken to exclude patients with severe SLE from the classification process. Subsequent studies will augment the sample size, enhance the portability and real-time performance of the detection platform, and validate the universality of the biomarkers. Furthermore, these studies will explore the use of spectroscopy combined with in-depth modeling in the early diagnosis of different diseases and activity levels. The combination of multimodal information will enhance the effectiveness of these approaches and drive precision medicine and large-scale health management forward.

## 5 Conclusion

In this study, a new classification and diagnostic model DBayesNet was proposed based on Raman spectroscopy combined with deep learning algorithms to be able to accurately differentiate between SLE and non-SLE populations in a fast and accurate classification method, which compensated for the shortcomings of poor classification accuracy due to the high overlap between spectral peaks and the difficulty of feature extraction in connective tissue diseases, and achieved a fast and accurate diagnosis of SLE patients, and meanwhile, spectroscopic analyses revealed that amide, proline and tryptophan may be the characteristic molecular biomaterials that differentiate the spectra of diseases., proline and tryptophan are likely to be the characteristic molecular biomaterials that distinguish the disease spectrum. Meanwhile, the two-branch Bayesian model achieved the best classification results in terms of sensitivity, specificity, precision, and accuracy of 91.6%, 80.0%, 82.3%, and 85.9%, respectively, compared with the traditional classification model. This study demonstrates that Raman spectroscopy combined with deep learning methods can help to understand the differences between SLE and non-SLE substances in serum in humans, which provides powerful technical support in the early diagnosis and activity assessment of SLE and also provides a useful reference for dealing with biomedical data at different scales, which is of great clinical application and research value.

## Data Availability

The raw data supporting the conclusions of this article will be made available by the authors, without undue reservation.
